# “If I’d Known …”—a Theory-Informed Systematic Analysis of Missed Opportunities in Optimising Use of Nicotine Replacement Therapy and Accessing Relevant Support: a Qualitative Study

**DOI:** 10.1007/s12529-018-9735-y

**Published:** 2018-07-30

**Authors:** Aleksandra Herbec, Ildiko Tombor, Lion Shahab, Robert West

**Affiliations:** Department of Behavioural Science and Health, University College London, Gower Street, London, WC1E 6BT UK

**Keywords:** Smoking cessation, Nicotine replacement therapy, Pharmacotherapy, COM-B, TDF, Qualitative study, Adherence

## Abstract

**Purpose:**

Nicotine replacement therapy (NRT) is often used suboptimally by smokers. Previous research has focused on cognitions and attitudes as potential reasons. This study drew on theoretical frameworks of behaviour to comprehensively explore smokers’ NRT use to identify new intervention targets.

**Methods:**

Semi-structured face-to-face interviews were conducted with 16 adult UK-based smokers and ex-smokers who used NRT in recent quit attempts (mean (SD) age = 34.9(10.3); 82.3% women). The COM-B (capability, opportunity, motivation, behaviour) model and the theoretical domains framework informed the interviews and analyses. Data were analysed in NVivo 11.

**Results:**

Two related behaviours were identified relevant to NRT use: use of NRT per se and engaging with information and support with NRT use. A meta-theme of “missed opportunity” identified instances when smokers did not or could not engage in these behaviours. For use of NRT per se, these included limited knowledge, poor technique of use, low motivation to optimise use, and lack of role models. For engaging with information and support, they included low awareness of optimal use techniques, selective information-seeking, low expectations, limited exposure to guidelines, deficient advice from healthcare professionals, and suboptimal product display. Prior suboptimal experience tended to negatively affect subsequent use and views. Participants were interested in accessible and comprehensive guidelines on NRT and its use.

**Conclusions:**

There appear to be important missed opportunities for optimal use of NRT both in terms of use itself and engagement with information on optimal use. These missed opportunities arise from a range of capability, motivational, and opportunity-related factors.

**Electronic supplementary material:**

The online version of this article (10.1007/s12529-018-9735-y) contains supplementary material, which is available to authorized users.

## Introduction

Nicotine replacement therapy (NRT) is the most commonly used medically licenced pharmacology for smoking cessation, although its use has been declining over the past decade and is now over taken by electronic cigarettes [1, 2]. Importantly, there is a disconnect between the observed effectiveness of NRT in clinical trials and that found when NRT is bought over the counter (OTC) [3–6]. Suboptimal use of medications, such as using too little or terminating treatment before the recommended 8 weeks, may be a plausible reason, and better use of NRT outside of research settings could improve its effectiveness [7]. However, there is little direct evidence to guide interventions to promote more effective use of NRT, especially among smokers purchasing it OTC [8, 9]. Attempts to understand suboptimal use have focused previously on cognitive and attitudinal factors (e.g. what smokers know and think about NRT), but it is possible that a wider range of factors might play a role [10, 11], for example the support received and the technique of application, as was shown to be the case with other medications [12]. This study explored these wider factors using a systematic and theory-driven method.

NRT comprises a broad range of medically licenced products that deliver nicotine that can help alleviate withdrawal symptoms during quitting [13]. NRT includes a slow acting nicotine transdermal patch (to be applied daily for 16 or 24 h) and fast acting nicotine forms, such as nicotine gum, lozenges, and sprays (to be applied as frequently as every 1–2 h). The different NRT products are similarly effective, meant to be used for 8 weeks, and have been shown to be generally safe, also for long-term use [4, 14–18]. Combination of the nicotine patch and a fast acting NRT is more effective than using single NRT products [3]. Many forms of NRT are available both on prescription and OTC in many countries, including in Europe, Canada, USA, and Australia [19, 20]. In the UK, all forms of NRT are available both on prescription (at no or lower cost) or for OTC purchase in pharmacies, supermarkets, and some local stores, and UK-based smokers can access stop smoking services that are free at the point of access [5].

However, effectiveness of NRT when bought OTC and used without professional support tends to be low [5]. Several explanations for this have been put forward. One is that the effect of NRT has been overestimated in meta-analyses due to a range of biases, e.g. industry funding, and thus the lower performance of NRT in the real-world might reflect more closely the medication’s true effectiveness [21, 22]. Another is poor adherence, with smokers using too little NRT, for too short a period of time, or possibly incorrectly [7, 23–25].

A number of factors have been shown to contribute to NRT non-adherence. A recent review of 48 studies examining correlates and self-reported reasons for suboptimal use of NRT and other cessation pharmacotherapy among smokers and ex-smokers [8] has proposed to distinguish between non-preventable factors (e.g. comorbidities, tobacco dependence, and sociodemographic characteristics) and preventable factors (e.g. beliefs, attitudes, and psychosocial characteristics). Several conceptual frameworks of non-adherence can also be applied to understand NRT use, including a broad distinction between intentional (e.g. not wanting to use medications) and non-intentional (e.g. forgetting) non-adherence [26, 27]. The Necessity-Concerns Framework [28] suggests that adherence is affected by implicit evaluations of medicines in terms of patients’ *perceived* need (e.g. importance of medication to improve condition) and side effects. The Attitudes Toward Nicotine Replacement Therapy scale [29] assesses similar attitudinal factors, as well as knowledge of NRT. Many quantitative and qualitative studies lend support for these frameworks, showing that misconceptions, concerns over safety, and low efficacy beliefs to be linked to NRT non-adherence [23, 29–40]. Interventions that target these constructs and include reminders to use medications or materials with educational and problem solving components have shown positive but limited impact on adherence and subsequent abstinence [9, 25, 34].

Therefore, to date, research has focused on assessing and addressing beliefs, attitudes, and knowledge of NRT, but little is known why smokers hold such views, how they behave when they initiate and use NRT, and how they interact with resources on NRT use [8]. In order to advance our understanding of NRT use as well as develop future interventions and clinical guidelines, there is a need for a more comprehensive assessment of this behaviour.

The COM-B model [41] offers a broad framework for understanding different behaviours (e.g. [42, 43]) and was also suggested as a basis for development of interventions targeting medication adherence [11], but it has not yet been systematically applied to NRT use. The COM-B model proposes that the following components interact in a dynamic way to produce a behaviour: capability (physical, e.g. skills, and psychological, e.g. knowledge), opportunity (physical, e.g. access, and social, e.g. support), and motivation (reflective, e.g. identify, and automatic, e.g. emotions). COM-B has been elaborated in some areas by theoretical domains framework (TDF) [44, 45]. The TDF resulted from a synthesis of 33 theories and 128 constructs [44] and consists of 14 domains (e.g. *skills*, *identity*), which can help to examine influences of behaviour, but it does not specify the relationship between the individual elements. TDF domains can be mapped onto the COM-B model (see Supplement 1), thus together creating a more detailed theoretical framework that can facilitate collection and analysis of data pertaining to behaviours and factors affecting them and identify the key barriers and facilitators [41].

Use of COM-B and the TDF may therefore provide a more comprehensive consideration of factors affecting NRT use than previous approaches. This in turn may help to identify novel opportunities for research as well as inform complex interventions and clinical practice relevant for treating tobacco dependence and NRT use [10, 41, 46].

### Aims

This exploratory study applied a theoretical framework informed by the COM-B model and TDF to understand suboptimal use of NRT by UK-based smokers, especially when obtained OTC, including capability and opportunity factors that have received less attention to date.

## Methods

### Design

The study was conducted as part of a larger research programme that aimed to understand what kind of support would be acceptable and potentially beneficial to smokers to optimise their use of NRT, with focus on OTC NRT. The present study involved in-depth semi-structured individual face-to-face interviews and was supplemented by a think aloud procedure about a prototype smartphone application (app) supporting NRT use (NRT2Quit). The app was developed to be evaluated in a separate study (ISRCTN33423896). The reporting follows the COREQ guidelines [47].

### Participant Recruitment

Convenience sampling was used, with participants recruited from the general population through online advertisements, mailing lists, posters around University College London, and word of mouth. Recruitment materials invited participants to an interview study as part of a project that aimed to develop aids and tools to support NRT use while quitting, including apps. To take part, participants had to (1) be 18 years old or older, (2) have used any OTC NRT products in the past 5 years as part of quitting, (3) be a current or recent daily smoker, or currently trying to quit, (4) own a smartphone and be interested in using apps, (5) be fluent in English, and have good or corrected-to-normal vision.

### Procedure and Interviews

The interviews were conducted in three phases (two in December 2014, nine in the summer of 2016, and five in the summer of 2017). There has been no substantial change to guidelines and access to NRT in this time period. The first two interviews initially prioritised usability testing of NRT2Quit - a new smartphone app supporting NRT use during quit attempts that was developed by the first author with support from other project members at UCL (see Supplement 2 for more information). However, emergence of important themes related to NRT use and context of NRT use motivated pausing and re-scheduling data collection to 2016, when an updated interview guide was used. The final five interviews were conducted after initial data analysis, as it was judged necessary to ensure that data saturation was reached [48, 49]. Participants were reimbursed with vouchers of £20 in 2014 and, due to extending the duration of the interviews, £30 in 2016–2017. The interviews were conducted on university campus in London.

As part of the study participants completed questionnaires on history of smoking and quitting, use of NRT, self-assessed knowledge of NRT, the type of support with NRT use they accessed, and satisfaction with the available support. The interviews were conducted by the first author and lasted 50–90 min. The interviews followed a semi-structured interview schedule (Supplement 3) and were divided into two parts. The first part was guided by the COM-B model and TDF and explored participants’ (i) experiences with NRT use, from initiation to termination; (ii) knowledge, skills, and views pertaining to NRT and its use; (iii) experiences with and views on the available support with NRT use; and (iv) preferences for support with NRT use, including digital support. The second part used the NRT app as a prompt and involved think-aloud methodology [50] to elicit views on (v) advice and recommendations on NRT use provided in the app (e.g. advice on use of combination NRT, i.e. patch and a fast acting form; data reported here), and on (vi) features offered within the app and suggestions for its improvement (data not reported here).

Participants’ responses guided the interview progression, but the interviewer ensured that all core topics were discussed. Impromptu questions were asked to elicit elaboration. During the interview, after participants described their accounts and views, the interviewer briefly clarified guidelines around NRT use or any uncertainties, particularly around safety, regimen, and combination NRT. Except for the NRT2Quit app, no other prompts were used. The interviews were audio-recorded and transcribed intelligent verbatim by a third party, who signed confidentiality agreements. Participants’ data was labelled with codes to protect their identity.

### Data Analysis

Interview transcripts were analysed using principles of framework analysis (FA) [51], which has been commonly used in applied health research [36, 52–54]. FA supports a transparent and systematic analysis of large volumes of qualitative data and is particularly suitable in projects with a well-defined participant sample and pre-determined themes, while also enabling emergence of novel themes [32]. FA involves: (i) *familiarisation* through reading and re-reading of transcripts, (ii) *identification of recurrent themes and subthemes* using pre-defined and new emerging codes, (iii) *development and refinement of a thematic framework* through systematic indexing of transcripts, and (iv) *development of descriptive accounts* and *creation of explanatory frameworks*. Since the current study was primarily exploratory, all participants’ statements were treated as potentially important and a realist epistemological perspective was adopted [35]. Data analysis was conducted in NVivo 11.

The first round of coding involved detailed indexing of all the data, with the labels identified both deductively from the interview guide and inductively. These labels were then grouped into themes within a coding framework that incorporated constructs from the COM-B model and TDF version 2 (Supplement 1) [41, 44, 45]. Data were coded to multiple codes and COM-B and TDF domains as relevant [44, 45, 55]. The final thematic framework was agreed through several iterations and internal validation [49] conducted by the first two authors. Additional codes were devised for data falling outside of the COM-B framework. These included data related to participants’ reactions to facts and guidelines on NRT use (also reported in this write up), as well as participant background (e.g. smoking profile, experience and views on digital programmes), data context (e.g. discussion of past experiences, preferences for support), and code “other” for all other content (e.g. discussion or procedures). We used constant comparison [35] and deviant case analysis [36] to ensure internal validity.

### Research Team and Reflexivity

The study was conducted by experienced mixed-methods researchers specialising in tobacco dependence and its treatment, behaviour change, and the development and evaluation of complex interventions using COM-B and TDF. The interviews were conducted by the first author—a PhD candidate specialising in health psychology, who had experience in conducting and analysing qualitative studies. The participants were informed that the interviewer was a member of the group that works on creating new stop smoking aids, including the app discussed at the interview. Participants were encouraged to share all the insights they were comfortable with and to be honest, as their accounts could inform future aids for quitting and medication use created by the group.

## Results

### Participants

The interviews were conducted with 16 adult participants (mean age = 34.9, standard deviation = 10.3) of whom 13 (81.3%) were women, 13 (81.3%) worked in non-manual jobs, and 11 (68.8%) are current smokers (see Table 1). Most participants had tried at least two different NRT products in the past (81.3%), most commonly the patch or gum (93.8%), and three had tried combination NRT (18.8%). Most (68.8%) participants had some prior experience with receiving support or advice with NRT use from different healthcare professionals (HCPs, e.g. pharmacy staff or General Practitioners, GPs), but satisfaction with the available support tended to be low (mean rating 2.5, SD = 0.8 on a scale 1–5).

**Table 1:** Characteristics of the interviewed participants

ID	Sex	Age range	Smoking status during the interview	CPD	Last quit attempt last year (vs. earlier)	Patch	Gum or lozenges	Sprays or inhalators	Combination NRT	Accessed any HCP’s support with NRT use	Satisfaction with the available support on NRT use (1 = not at all, 5 = completely)	Ratings of knowledge on NRT, regimen and application techniques (1 = none, 5 = very good)		
												NRT	Regimen	Technique
P1	f	18–34	Quit	1–5+	Yes	–	Yes	Yes	–	Yes	2	3	2	2
P2	f	18–34	Daily	5	–	Yes	Yes	–	–	–	2	2	2	2
P3	f	18–34	Daily	15	Yes	Yes	Yes	Yes	Yes	Yes	3	4	3	4
P4	f	18–34	Daily	5–10	Yes	–	Yes	–	–	Yes	2	3	3	3
P5	m	35–70	Quit	np	–	Yes	Yes	Yes	Yes	Yes	2	2	2	3
P6	m	18–34	Daily	8–10	Yes	Yes	Yes	Yes	–	Yes	4	4	4	4
P7	f	35–70	Non-daily	10–12	Yes	Yes	Yes	Yes	Yes	Yes	2	3	2	2
P8	f	35–70	Daily	20	Yes	Yes	Yes	Yes	–	Yes	3	2	2	3
P9	f	18–34	Daily	25	Yes	Yes	Yes	Yes	–	Yes	1	2	3	2
P10	f	35–70	Daily	6–7	Yes	Yes	Yes	–	–	Yes	3	4	3	3
P11	f	35–70	Daily	5–10	Yes	Yes	Yes	Yes	–	–	3	4	5	4
P12	m	35–70	Quit	9	Yes	–	Yes	Yes	–	–	3	4	3	4
P13	f	35–70	Non-daily	20	Yes	Yes	Yes	Yes	–	–	3	3	2	2
P14	f	35–70	Non-daily	3	–	–	Yes	–	–	–	1	2	2	2
P15	f	18–34	Quit	20	Yes	Yes	Yes	Yes	–	Yes	3	3	2	2
P16	f	18–34	Daily	10–15	Yes	Yes	–	–	–	Yes	3	2	5	1

f = female; m = male; CPD = cigarettes smoked per day when smoking; HCP = healthcare professional, man-manual employment, n-man = non-manual employment; np = not provided; NRT = nicotine replacement therapy;

### Overview of Qualitative Findings

The first round of analysis resulted in a single COM-B- and TDF-informed coding framework that captured all the interview data. However, through further iterations the analysis supported distinguishing between two inter-related behaviours emerging from the data, which were subsequently analysed using separate, parallel thematic frameworks informed by COM-B and TDF. The first behaviour (B1) was “using NRT per se,” and the second behaviour (B2) was “engaging with support and resources on NRT use.” Table 2 reports higher-order themes and subthemes for each of these behaviours. A summary of findings related to each of the two behaviours and COM-B domains is reported below. Illustrative quotes for each COM-B domain are reported in Tables 3 and 4 for B1 and B2, respectively.

**Table 2:** Thematic framework informed by the COM-B (capability, opportunity, motivation, and behaviour) model and Theoretical Domains Framework for two behaviours: using NRT per se (B1) and engaging with information and support on NRT use (B2)

COM-B	Using NRT per se (B1)	Engaging with information and support with NRT use (B2)
B	1. B1: Using NRT per se—from imitation to termination 1.1 Product selection and initiation of NRT use 1.1.1 Formal health care channels - GP recommendations or prescription - Recommendations in pharmacies - Stop smoking programmes - Samples from healthcare professionals 1.1.2 Informal channels - Word of mouth - Internet, TV and other advertisements - Samples from acquaintances 1.2 Selection criteria for NRT type 1.2.1 Convenience 1.2.2 Level of addiction and NRT strength 1.2.3 Prior experience 1.2.4 Cost, flavour and other criteria 1.2.5 Spontaneous and unguided selection 1.3 Experience of purchasing NRT 1.4 Past NRT use 1.4.1 Using individual NRT 1.4.2 Using multiple NRT products 1.4.3 Adhering to guidelines and recommendations 1.4.4 Experiencing side effects 1.5 Termination of NRT use	13. B2: engaging with information and support on NRT use 13.1 Using NRT without any support 13.2 Reliance on one’s experience and understanding of addiction 13.3 Engaging with patient information leaflets 13.3.1 Reading the leaflet 13.3.2 Selective reading 13.3.3 Ignoring the leaflet 13.4 Engaging with healthcare professionals 13.4.1 GPs 13.4.2 Cessation advisers 13.4.3 Pharmacists and pharmacy staff 13.5 Accessing informal sources of support and information 13.5.1 Friends and family 13.5.2 Internet 13.5.3 TV and other ads 13.6 Engaging with display and packaging of NRT
C Phys	2. Physical skills in taking NRT 2.1 Techniques and application methods 2.2 Practice and experimentation with NRT use	Not identified as a theme
C Psy	3. Knowledge related to NRT use 3.1 Factual knowledge about NRT 3.1.1 Types of NRT 3.1.2 Combination NRT 3.1.3 Mechanisms of action and ingredients 3.1.4 Effectiveness 3.1.5 Safety and side effects 3.2 Procedural knowledge about NRT use 3.2.1 Knowledge of techniques and application methods 3.2.2 Regimen of NRT use 3.3 Misconceptions and factual errors	14. Knowledge of sources of information and support with NRT use
	4. Memory and attention to take NRT 4.1 Remembering about NRT use 4.2 Competing tasks and attention to NRT	15. Memory and attention for information and support on NRT use 15.1 Focus on potential harm and side effects 15.2 Limited attention and recollection of advice
	5. Behaviour regulation in NRT use 5.1 Mental stamina to endure negative sensations 5.2 Monitoring and scheduling NRT use 5.3 Planning and preparing for obtaining NRT	Not identified as a theme
O Phys	6. Physical opportunity for NRT use 6.1 Views on NRT products in general 6.1.1 Range of NRT products 6.1.2 NRT product design 6.1.3 NRT cost and availability 6.2 Views on individual NRT products 6.3 NRT regimen 6.3.1 Dose recommendations 6.3.2 Combination NRT 6.3.3 Cognitive complexity of NRT regimen 6.3.4 Impracticality, convenience and high effort 6.4 Views on medications and pharmaceutical companies	16. Physical opportunity for engagement with information and support 16.1 Pharmacy setting 16.2 Display and packaging of NRT 16.3 Views and preferences on current printed resources on NRT use 16.3.1 Accessibility of guidelines 16.3.2 Patient leaflets 16.3.3 Other written resources 16.4 Digital support with NRT use 16.4.1 Online resources 16.4.2 Smartphone apps
O Soc	7. Social opportunity and perceived norms impacting on NRT use 7.1 Role models in relation to NRT use 7.2 Use of NRT products in public	17. Social opportunity 17.1 Not being offered support from healthcare professionals 17.2 Views and preference regarding face-to-face support 17.2.1 Accessibility of support 17.2.2 Dissatisfaction with past support 17.2.3 Detailed consultation 17.2.4 Signposting 17.2.5 Support in the pharmacy 17.2.6 Anonymous support 17.3 Peer testimonials and demonstrations
M Ref	8. Beliefs about capabilities to use NRT	Not identified as a theme
	9. Beliefs about consequences of using NRT and other medications 9.1 NRT effectiveness 9.2 NRT safety concerns 9.2.1 NRT and addiction 9.2.2 Overdosing and dual use with cigarettes 9.2.3 Side effects 9.2.4 Other concerns 9.3 Views on smoking and quitting that could impact on NRT use 9.3.1 Quitting requires commitment and willpower 9.3.2 Smoking as a habit and learned gestures	18. Beliefs about consequences of engaging with information and support on NRT use 18.1 Value of accessing support 18.1.1 Face-to-face support 18.1.2 Self-help resources 18.1.3 Reliance on one’s experience and knowledge 18.2 Burden of commitment to face-to-face support 18.3 Right timing and frame of mind needed for commitment to quitting
	1. Identity related to NRT use	Not identified as a theme
M Aut	2. Emotions: anxiety related to NRT use	19. Emotions: shame and embarrassment to engage with support
	3. Routines and habits in NRT use	
–	20. Reaction to NRT facts and recommendations 20.1 Shock and surprise 20.2 “aha” moment—re-assessing one’s prior knowledge and experiences with NRT 20.3 Feeling encouraged 20.4 Ambivalence	

B = behaviour; C Phys = capability (physical); C Psych = capability (psychological); O Soc = opportunity (social); O Phy = opportunity (physical); M Ref = motivation (reflective), M Au = motivation (automatic), GP = in the UK general practitioner (e.g. primary care physician in the US)

**Table 3:** Illustrative quotes for each domain of COM-B (capability, opportunity, motivation and behaviour) for behaviour 1 (B1): use of NRT per se

B1: Use of NRT per se	
“[The adviser] gave me just a couple of packets of gum [and] the mints, just to try, but I never went beyond […] But because she did not give it to me in [a normal package] then I did not get [instructions]” (P1)	
“I just grabbed the strongest one [inhalator] that they had out there because I smoked a lot” (P15).	
“I just thought “Oh if the gum is rubbish, everything else will be rubbish,” so I will not try anything else.” (P14)	
B1: capability (physical) to use NRT	
“I chewed it as a normal gum […] I had no idea [there was a special technique for gum use], no wonder I thought it was gross.” (P14)*	
“Yeah, the inhaler, the little white one I used to try and smoke it like it was a cigarette.” (P3)*	
“I just saw it [gum] in Boots or Superdrug and thought I’d give it a go because the patches were not working and I just thought it tasted disgusting so I just, you know, that was, it was one time I tried it […] I kind of just wrote it off.” (P2)	
B1: capability (psychological) to use NRT	
“[Combination NRT] goes against what I read twenty, you know, […] many, ten [years ago].” (P13).	
“[NRT patch placed on torso] is going directly into your bloodstream and […] it’s near to the vital organs I suppose, so I felt more, had a more problem with that, yeah.” (P5)*	
“It was just when I was having an immediate craving, I would have a gum then. I think after eating as well, that’s a good time and it was fine but it was like it was just never enough.” (P3)*	
B1: opportunity (social) to use NRT	
“[Using inhalator among friends made me feel] Like a bit of an idiot really. […] it looks a bit like a tampon holder or something” (P1)	
“I think it would be nice to have the information on a website that I could find or go to a forum and read about people’s experiences with it.” (P2)	
“I would have tried it if I’d had the, I guess the reading material and the advice and proven that it had helped somebody else I would have done it but I did not have any of that, so I just left it all.” (P14)	
B1: opportunity (physical) to use NRT	
“that was annoying as well, being told not trying get it [patch] wet and trying to position myself in the shower for it, it didn’t work.” (P9)	
“I just think [taking NRT] five to ten times a day is a lot. […] People’s going to forget when they get busy.” (P6)	
“[NRT should be] something portable, easier to remember, and cheaper than tobacco as well.” (P11)	
B1: motivation (reflective) to use NRT	
“I just thought it was not working at all and I still wanted to smoke so I just threw it away.” (P14)	
“Each one [quitting method] is more suited like to other people, like some are more suited for the patch, or the gum, or whatever, or just willpower.” (P6)	
“I didn’t like [the patch] ‘cos I thought people were looking at me knowing that I smoke […] and that I’m some kind of addict” (P9)	
B1: motivation (Automatic) to use NRT	
“So that was one of the other things that made me nervous a bit of this gum because I thought “oh gosh, what if I become addicted to the gum […] I was worried that I was going to kind of, you know, give myself nicotine poisoning.” (P4)*	
“Obviously you didn’t see it [the patch] when it was covered up but when it wasn’t covered up and there were hot days, like recently I’ve felt horrible, I felt a bit embarrassed almost” (P9)	
“I mean I work but he did give me some so you know, I was buying them and then I just fell out of the pattern of buying them and then ran out of them and then ending up smoking” (P10)	

*Quotes indicating misconceptions about NRT and application techniques

**Table 4:** Illustrative quotes for each domain of COM-B (capability, opportunity, motivation and behaviour) for behaviour 2 (B2): engaging with information and support with NRT use

B2: behaviour: engaging with information and support with NRT use	
“The lozenges I kind of knew what [the leaflet] was going to say […] you can work that one out […] I did read the gum advice and I did read the patches advice at some point, […] anything else I haven’t because I kind of know how it works” (P13)	
“No, I didn’t [get advice from a pharmacist], when they’d say ‘do you know what, like have you used it’, I’d say ‘yes’, because I don’t like, because I always feel like you’re going to just end up getting advice and then feel guilt-tripped into it!” (P15)	
“On Amazon I just looked at reviews and it had like four point some rating out of five so people said it was helping them so I mean that’s why I tried it, give it a shot.” (P2)	
B2: capability (psychological) to engage with information and support with NRT use	
“I did not actively go for a technique, search for a technique but that’s because I did not know there was a technique.” (P12).	
“No, I only read the side effects [on the leaflet]” (P16)	
“We all, a lot of our questions to begin with was what happens if we smoke a cigarette and we are wearing a patch? Or use the inhalator than have a cigarette.” (P8)	
B2: opportunity (social) to engage with information and support with NRT use	
“I just used to take it up to the counter and that’s fine, no one ever said “We have these options” or “Have you tried this programme or there’s...?” No, nothing like that.” (P14)	
“I was in a [cessation] group this year […] I promise you, it [combination NRT] was definitely not [mentioned]. no way were any of us told, honestly, that if you take two together, no.” (P8).	
“You feel like a failure when you’ve like relapsed so if you actually had more information about how to take things properly maybe it would have better chances.” (P3)	
B2: opportunity (physical) to engage with information and support with NRT use	
“If it was pharmacy based a lot of people think, you know, I’ve got queue, got to talk, got to get questions and wouldn’t bother.” (P10)	
“I don’t believe for a minute that [pharmaceutical companies] have optimised the information [patient leaflets] for customers.” (P13)	
“I only bought what I had initially which was the one with the green tab which is what I remembered but when I went there was like lots of stuff. I was like wow, it’s a big range […] it’s really quite shocking.” (P10)	
“Then there are dozens of other things that you need to prioritise […] so if there is a technique I think it should be communicated in a very small amount of time and when the other person’s attention, whether it’s at doing an advertisement or at the point of sale.” (P12)	
“I think if someone had said to me, “Do you know about this, you know, this leaflet of information or the support that you could get from your doctor or even an app,” I would have used it.” (P14)	
B2: motivation (reflective) to engage with information and support with NRT use	
“No, I just thought that I could just do it, I just thought it was just straightforward.” (P7).	
“And then you feel like you have to commit to it properly because someone’s helped you. […] it’s just the medical environment does feel intimidating in many ways and you just feel like you have to, you know, like you have to commit to this and like someone’s going to be checking up on you to make sure that you’re actually doing it.” (P15)	
“In the end you actually write those instructions for yourself, because it has to be tailor-made for you, because what they put on the instructions is a generic, but not one shoe fits all.” (P7).	
B2: motivation (automatic) to engage with information and support with NRT use	
“I was just too embarrassed, so I just went in and grabbed some gum and thought “I’ll try this” and I didn’t even really look into it.” (P14)	
“And like if you go back to the same person each time you might feel like a bit embarrassed, so if there was like anyone that you could go up to at any time point, that might be helpful.” (P3)	
“People are more intimidated like when it’s like a doctor or a pharmacist because, again, you just feel like you know, you have to.” (P15)	

Additionally, each of the coding frameworks included a meta-theme and associated subthemes related to “*Missed Opportunities*”—instances or circumstances identified by the authors as preventing smokers from taking full advantage of the available resources or to otherwise optimise the target behaviour. These included challenges, barriers, or shortcomings, also in light of best clinical practice or evidence-base in smoking cessation, and thus could constitute relevant targets for future interventions. These missed opportunities are reported in Box 1 for each of the two behaviours. Finally, we also report data from a theme capturing participants’ reactions to the guidelines and recommendations for NRT use that were discussed during the interview.

Box 1 Summary of missed opportunities in capability, opportunity, and motivation in using NRT per se (B1) and in engagement with information and support with NRT use (B2)

**Table:** 

Missed opportunities in using NRT per se (B1)	
Key challenges • Inadequate process of NRT selection • Suboptimal use of NRT Capability to use NRT • Limited knowledge of recommended application techniques • Limited knowledge of regimen of individual and combination NRT • Incorrect application of NRT • Misconceptions and factual errors that negatively impact on NRT use • Poor behaviour regulation: limited planning, scheduling, monitoring and stocking on NRT supplies • Low acceptability and limited endurance of unpleasant sensations and side effects Opportunity to use NRT • High NRT cost • Unattractive and impractical product design • Complex and burdensome NRT regimen • Lack of appropriate role models for NRT use Motivation to use NRT • Low motivation to optimise use • Limited expectations and uncertainty of benefit from NRT use • Concerns over safety and side effects • Negative beliefs and emotions, including anxiety when using NRT • Negative identity of NRT users, associating NRT use with greater addiction and desperation • Unhelpful beliefs about smoking, addiction, quitting, and medications in general	
Missed opportunities in engagement with information and support with NRT use (B2)	
Key challenges • Insufficient engagement with resources and face-to-face support on NRT use • Over-reliance on prior experience and informal sources of information Capability to engage with support on NRT use • Low awareness of the intricacies of NRT use that require additional information and support • Low awareness of guidelines and techniques that could help optimise NRT use • Preoccupation with information on potential harm, rather than on optimisation of use Opportunity to engage with support on NRT use • Limited access to and exposure to comprehensive guidelines on NRT use • Unattractive patient leaflets • Deficient advice and support offered by healthcare professionals • Busy pharmacy environment • Overwhelming and uninformative NRT product display Motivation to engage with support on NRT use • Limited expectations to benefit from resources and support on NRT use • Embarrassment to seek face-to-face support • Low acceptability of face-to-face support and anticipated commitment	

### B1: Behaviour of “Using NRT Per Se”

Participants obtained NRT in a range of contexts in the past, primarily buying it OTC, but a few received some of their NRT as part of cessation programmes, and through being offered samples from friends or HCPs, often without further guidelines. Although some participants knew about the wide range of NRT and believed that individual products could suit different preferences and circumstances, only few participants described systematic selection of NRT. Often the selection was spontaneous, or informed by prior experience, advertisement, or word of mouth. Strength of NRT and perceived convenience of the NRT, especially for the patch, were important selection criteria.

Most participants experienced side effects, and few reported benefitting from NRT use, which were all reported as reasons for terminating NRT use. Poor experience with NRT seemed to undermine efforts at establishing a routine for medication use, but the latter also contributed to forgetting and poor adherence. Additionally, negative prior experience with NRT tended to discourage future use of NRT products.

#### B1: Capability to Use NRT

Participants had some confidence in their knowledge about using NRT (also see Table 1), but their actual knowledge was limited and often included misconceptions with regard to NRT products, mechanisms of action, guidelines for use (including combination NRT and specific techniques of application), effectiveness, and safety. This was also the case with participants who accessed specialist cessation support before.

Prioritising and remembering to take NRT was challenging, especially amidst busy daily routines. Some participants realised they would need to set up routines or reminders (e.g. apps) to use NRT regularly. However, participants rarely discussed efforts to ensure adequate supply of NRT, or scheduling or monitoring its use. Indeed, participants tended to use fast-acting NRT (e.g. gums, sprays) when experiencing cravings or in situations when they would normally have a cigarette, rather than at scheduled or regular times. They also had difficulties persisting with NRT use when experiencing side effects.

Finally, participants’ accounts of NRT use suggested that many of them lacked skills to correctly use or apply these products. Practicing and experimenting with NRT use to improve effectiveness or to minimise side effects were very uncommon.

#### B1: Opportunity to Use NRT

Some participants were happy about the range of NRT available, but many thought the products were expensive. The design of some NTR products was judged unattractive, and participants felt uncomfortable or even embarrassed to use some of them in public (e.g. the inhalator). Additionally, the recommended regimen for NRT use, including combination NRT, was considered complex, inconvenient, effortful, and potentially harmful. Moreover, interviewees also lacked positive role models and access to success stories of NRT use that could encourage and guide them. Finally, some participants held unfavourable beliefs on medications in general that also affected NRT use.

#### B1: Motivation to Use NRT

Participants had confidence in their use of NRT and demonstrated motivation to initiate NRT use, often purchasing it OTC. However, they were not necessarily motivated to continue using it, or to improve NRT use. Only few participants found using NRT helpful during quit attempts, and many were unsure or had low expectations to benefit. Safety concerns were common, especially around over-dosing nicotine, which triggered anxiety.

Certain beliefs about smoking and quitting and particularly perceiving smoking as a habit or set of learned gestures, or viewing quitting as an individual journey that requires personalised approach or mainly willpower to succeed, were also related to lower motivation to use NRT, as the latter was not seen as sufficiently important or relevant for quitting. Other barriers to using NRT and especially combination NRT were negative identity of an NRT user and associating NRT use with greater addiction or desperation.

### B2: Behaviour of “Engaging with Information and Support with NRT Use”

Many participants reported no information seeking, used NRT without support, or engaged only with informal sources of information (e.g. discussions with friends). Also, many participants never read patient leaflets, while those purchasing OTC NRT through pharmacies or shops tended not to browse through the products. Decisions regarding NRT selection and use were often based on participants’ understanding of their addiction to cigarettes, information in NRT advertisements and the internet, word of mouth, and prior experience with NRT products. Participants who were offered some assistance with NRT use by pharmacy staff at the time of purchase tended to decline it. Only few participants were actively seeking advice on NRT and its use from their doctors or other HCPs.

#### B2: Capability to Engage with Information and Support with NRT Use

Participants tended not to be aware of the intricacies of NRT use and the existence of more comprehensive and updated guidelines relating to NRT use, which in turn did not promote information seeking. They also often mentioned difficulties remembering the advice provided by HCPs. Participants who sought information or advice and, for example, read patient leaflets, tended to focus on the side effects, and potential harms from overdosing or dual use of NRT and cigarettes, rather than on how to optimise NRT use.

#### B2: Opportunity to Engage with Information and Support with NRT Use

Participants’ accounts suggested few opportunities to engage with relevant or comprehensive support with NRT use. Participants often described not being offered advice when purchasing NRT. Additionally, face-to-face appointments dedicated to quitting were viewed as scarce and difficult to schedule, while the pharmacy environment was seen as too busy to engage in a comfortable conversation. Some participants were accepting that pharmacy staff does not offer additional advice on NRT use. However, many participants were not satisfied with the level and quality of support available to them, and in the hindsight, felt it might have negatively influenced their NRT use. Additionally, it seemed that to some participants, the busy displays of NRT products in stores and pharmacies are overwhelming and do not encourage browsing. Finally, some participants expressed mistrust toward medication manufacturers and the advice provided by them in the patient leaflets.

Many participants expressed the need for having accessible, relevant, and comprehensive advice on NRT use, including broader information campaigns to inform smokers about any updated recommendations. Some had preferences for receiving such advice in the form of testimonials from other smokers experienced with NRT use, while others expected to receive advice from HCPs. Additionally, participants suggested that relevant advice should be provided already during product advertisement or sale. Finally, participants had little experience with digital cessation aids, including apps. Nevertheless, in this context of scarcity of readily available or acceptable guidelines, participants were receptive to the idea of having digital support with NRT use. However, they expected more comprehensive cessation support, rather than an app focused only on NRT use.

#### B2: Motivation to Engage with Information and Support with NRT Use

Most participants had low motivation to seek information or support with NRT use. On one hand, some participants viewed NRT as a simple product, had high perceived self-efficacy for NRT taking, and did not expect that medication use could be improved. On the other hand, many participants had low awareness of the existence of relevant support and guidelines and its potential value. Sometimes, this made it also difficult for participants to appraise during the interview the support they had received in the past or to suggest improvements to it. Additionally, participants anticipated that face-to-face support would require too much commitment or be embarrassing. Finally, some felt that generic guidelines are not beneficial to individuals.

#### Reactions to NRT Facts and Recommendations

During the interview and exploration of the NRT app, some participants were surprised about certain guidelines on NRT use, particularly, the recommended method of applying NRT or using combination NRT. Others experienced “*aha*” *moments* as they re-assessed their knowledge and prior experience with NRT, or their prior engagement with existing sources of information and support. Some participants became interested in trying out more effective ways of using NRT in the future.

… even from finding out a little bit more information about the fact that some NRTs I haven’t been taking them properly and there’s like different ways to use them from what I was thinking, I think that’s already made me feel a bit more positive! (P3)

## Discussion

In this study, two inter-related behaviours emerged that could help explain and which contribute jointly to the use of NRT especially that purchased over-the-counter: using NRT per se and engaging with information and support with medication use. Applying the COM-B model and TDF revealed some factors that had not been identified previously.

The study identified a range of missed opportunities directly related to using NRT, which could be addressed as part of complex interventions. Echoing previous research, the findings revealed important limitations in reflexive motivation and psychological capability, including misconceptions regarding NRT, its effectiveness and safety, as well as the benefits of using combination NRT, all of which are likely to negatively impact NRT use [8, 23, 29–40]. Additionally, the findings suggest dissatisfaction with NRT product design and overly complex regimen. Moreover, significant shortcomings in physical skills and procedural knowledge on NRT use were common across accounts, all of which may be contributing to avoidable side effects and poor effectiveness. Finally, the lack of role models for NRT use and perceived low acceptability of using NRT in public may constitute additional important, but still under-researched barriers. Interventions addressing these missed opportunities in NRT use should therefore incorporate behaviour change techniques (BCTs) that support shaping knowledge (individual BCTs 4.1–4.4) and comparison of behaviour (BCTs 6.1–6.3) [56], as well as utilise testimonials of smokers and ex-smokers with experience of NRT use.

Although guidelines and best practice on NRT use exist, important barriers emerged in smokers’ capability, motivation, and opportunity to engage with relevant information and support. Among important missed opportunities identified were as follows: low awareness of intricacies of NRT use and of available advice, low expectations to benefit from the available support, and pre-occupation with information related to harm and side effects, instead of advice on how to optimise use. Moreover, participants’ accounts suggest that the support available to them may not be comprehensive and up-to-date, even when it is offered by HCPs. Indeed, in recognition of the intricacies in NRT use, the UK National Centre for Smoking Cessation and Training (NCSCT) has developed a dedicated advanced online course for smoking cessation specialists that addresses many issues with NRT use. However, as less than 5% of UK smokers access stop smoking services [57], a large proportion of smokers may not have an easy access to appropriate advice. Moreover, prior research found that patient information leaflets for different medications fail to meet the needs of patients [58]. Poor engagement with and low perceived relevance of NRT patients leaflet were also a prominent theme in this study. Finally, the busy pharmacy environment and poorly organised NRT displays may also not be conducive for help- and information-seeking among smokers. This is also in line with research showing numerous barriers faced by pharmacy staff, including lack of training, resources, and space, to deliver appropriate cessation support to smokers [45].

Taken together, these findings elucidate numerous under-researched reasons for the limited knowledge, negative attitudes, and suboptimal use of NRT found in earlier studies [23, 29, 31] and highlight the need for more accessible, attractive, and comprehensive support with NRT use. Better-quality support could be expected to improve adherence, medication effectiveness, and thus also cessation outcomes [7].

### Strengths and Limitations

First, being a qualitative study among a relatively small and self-selected sample of smokers, these findings have limited generalisability. However, the sample size was adequate for an exploratory interview study [59], and data saturation was reached [60, 61]. Second, the study explored experiences with NRT use, some of which could had taken place several years before the interviews, and which could be affected by recall bias. However, even the more distant experiences seemed to still have an impact on views on NRT and its potential future use. Third, the use of the NRT app and discussion of guidelines on NRT use during the interview contributed to revealing important insights, but this methodology could be improved in the future by using additional standardised prompts, e.g. patient leaflets, photos of NRT display, and specific statements from the guidelines. Finally, the sample was predominantly female, had completed education beyond 16 years of age, worked in non-manual employment, owned a smartphone, and had motivation and possibility to purchase OTC NRT. Therefore, the issues and challenges identified in this population are likely to be even more prominent among smokers with lower socioeconomic status or who have more limited access or lower motivation to initiate NRT use.

### Future Research Directions

Many of the missed opportunities identified in this study have not been addressed in previous research and should be explored further in qualitative and quantitative studies. It would also be important to assess the prevalence of the factors identified in this study as contributing to suboptimal NRT use and engagement with support on NRT use among smokers in the UK and other countries. There is also a need for more research into effectiveness and acceptability of different versions of brief advice provided by pharmacy staff at the points of sale and creating acceptable standardised resources to offset any shortcomings in the advice offered by HCPs, who are recommending or selling NRT; as well as for creating and assessing new NRT product displays, packaging or patient leaflets that include, or draw attention to, best-practice and evidence-based advice.

### Implications for Clinical Practice

Smokers provided with NRT OTC or on prescription but without appropriate skill training and advice may be using it incorrectly, which is likely to cause side effects and lower effectiveness. Provision of NRT should always be accompanied by appropriate advice that focuses on instruction on use and reassures smokers about the recommended frequency and amount of NRT use, and one that addresses any previous experiences with the medications, which might have been negative. The findings also suggest that smokers may be particularly receptive to accessing other smokers’ testimonials and multimedia that support skills training.

One of the encouraging findings was that participants tended to be positively surprised by the recommendations on NRT use and were encouraged to use the medications better in the future, which was observed previously as well [39]. Nevertheless, this study suggests that smokers may be reluctant to engage with HCPs or printed materials. Future interventions may need to emphasise novelty and relevance of the advice to catch smokers’ attention and engage them.

Furthermore, the study revealed tendencies of smokers to generalise their limited knowledge and even distant negative experiences with individual NRT products to the others, often with detrimental impact on future use. Addressing these challenges may require a broader information campaign that draws on principles of making every contact count [62], runs across multiple channels, and which engages smokers at different points of contact, including not only during product advertisement but also through packaging, display, and at points of sale.

### Conclusions

The use of NRT by some smokers, not only when purchased over-the-counter but also with HCP’s support, is characterised by missed opportunities in terms of capability, motivation, and opportunity, both in relation to NRT use per se and accessing and using information about NRT. Interventions to optimise NRT use will need to address all of these.

## Electronic Supplementary Material

ESM 1 (DOCX 203 kb)
