# Supplementary material for: “If I’d Known …”—a Theory-Informed Systematic Analysis of Missed Opportunities in Optimising Use of Nicotine Replacement Therapy and Accessing Relevant Support: a Qualitative Study
Source: Int J Behav Med. 2018 Jul 30;25(5):579–91. doi: 10.1007/s12529-018-9735-y (PMC6182503; doi:10.1007/s12529-018-9735-y)
Supplement: Supplementary file 1 — (DOCX 203 kb) [file 12529_2018_9735_MOESM1_ESM.docx]

**Supplementary Material 1: The COM-B system and Theoretical Domains Framework (TDF)**

According to COM-B Model^[[1]](#footnote-1)^, for any behaviour to take place the individual has to have the physical and psychological capability, the physical and social opportunity (factors that are beyond the individual), as well as reflective and automatic motivation (different brain processes that ‘energize and direct’ the actions).

**Table S1: Mapping of COM-B and Theoretical Domains Framework (TDF, Version 2^[[2]](#footnote-2)^) that was used as a coding framework during the later phases of the interview data analysis.**

| **COM-B** | **Theoretical Domains Framework (TDF)** |
| --- | --- |
| **B – Behaviour** |  |
| **Capability -Physical** | 1. **Skills** |
| **Capability - Psychological** | 1. **Knowledge** |
|  | 1. **Memory, attention and decision making** |
|  | 1. **Behaviour regulation** |
| **Opportunity -Physical** | 1. **Environmental Context and Resources** |
| **Opportunity - Social** | 1. **Social Influences** |
| **Motivation - Reflective** | 1. **Beliefs about capabilities** |
|  | 1. **Beliefs about consequences** |
|  | 1. **Optimism** |
|  | 1. **Social/professional role and identity** |
|  | 1. **Goals** |
| **Motivation - Automatic** | 1. **Intentions** |
|  | 1. **Emotions** |
|  | 1. **Reinforcement** |

**Supplementary Material 2: NRT app used as a prompt– screenshots and additional information**

Below are screenshots of the app prototype used in the interview study (NRT2Quit). The NRT2Quit smartphone app was developed by the first author (AH) and other collaborators from the University College London to support the use of nicotine replacement therapy (NRT) during quit attempts. The app was informed by evidence, best clinical practice in the UK, and theory (COM-B and TDF). The app was used during the final stage of the interview as a prompt to discuss digital support with NRT use, and the specific guidelines and recommendations on NRT use.

The core functions in the app were: advice on individual and combined NRT tailored to the products purchased by smokers, monitoring of progress and feedback on behaviour (medication use and quitting), as well as tips on quitting smoking and craving management.


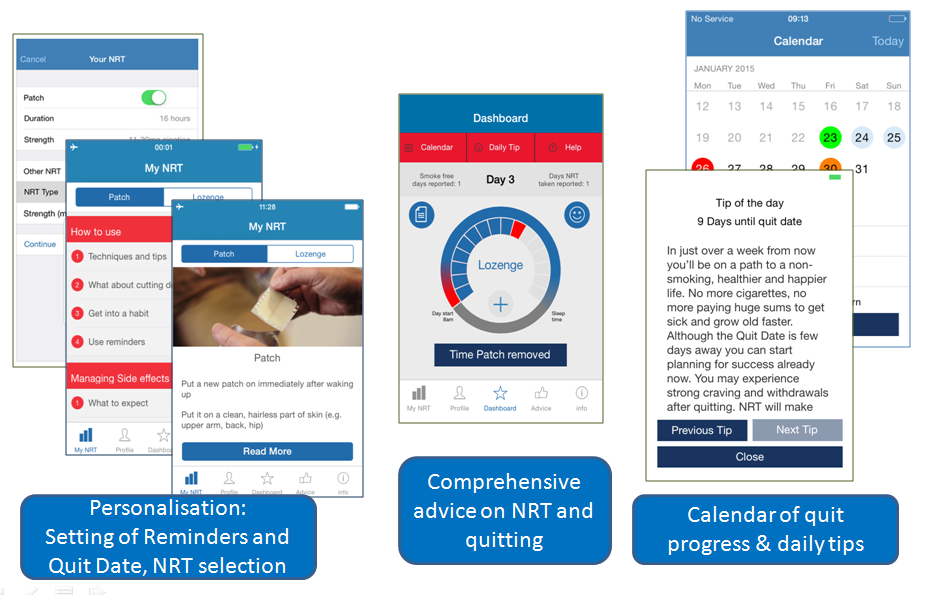


Funding: The cost of developing NRT2Quit was covered by a grant from the Global Research Awards for Nicotine Dependence (GRAND, 2013) Program funded by Pfizer, of which the principal investigator was Prof Tobias Raupach. The funding bodies had no role in the study or app development. NRT2Quit was evaluated in a separate study (ISRCTN33423896).

**Supplementary Material 3: Interview guide**

***Part 1 – exploring behaviours of NRT use and accessing support, and factors affecting these behaviours (informed by COM-B model)***

1. What are your experiences with quitting smoking to date?

- What kind of support have you used to quit smoking, if any?

1. What are your experiences with using stop smoking medications/NRT?

- Tell me about the nicotine products you’ve used. How did you use them?
- What has motivated you to use these products? How did you decide on them?
- What did you know / think about these products?
- How did you find using these products? What difficulties did you have?

1. What kind of support or help did you get with using these medications?

- Where/How/Why have you looked for information about NRT and using it?
- Did you speak to anyone about choosing or using NRT? Why? Why not?
- Was there anything that you found helpful? Not helpful? Why?
- What information or support with NRT taking would you like to have received?
- What would need to change for you to use NRT according to guidelines?

1. What are your experiences and views on using computer, online, or mobile programmes for quitting smoking?

- What would you expect from such a programme?
- How do you think such a programme could help you with medication taking?
- What would you expect from an app that aims to help with quitting with NRT?

***Part 2 - Views on the prototype app and advice within the app (think-aloud methodology)***

*Note: from among the emerging data, only the data relevant to COM-B analysis of the target behaviours: NRT use per se, and engagement with support and information on NRT use, are reported in this manuscript.*

*Task: “We are developing a novel smartphone app for smokers who want to use stop smoking medications - nicotine replacement therapy to quit smoking. In the first instance, the app would be available to people who want to buy nicotine replacement therapy without a prescription. I would like to get your views on the app in general, and also on the specific features and advice within the app. Please use it, and share any thoughts or comments out laud.”*

Participants were free to explore the app and comment on different features and content. Prompts and supportive questions included:

1. What are your initial thoughts about it in general / the layout / looks / information included?
2. What do you think about the advice provided?
3. What do you like/dislike about it?
4. What features do you think are most/least useful?
5. How do you feel when using the app?
6. Is there anything that should be changed / explained better / presented better?

1. Michie, S., M.M. van Stralen,R. West, The behaviour change wheel: a new method for characterising and designing behaviour change interventions. Implementation Science, 2011;6:42. [↑](#footnote-ref-1)
2. Atkins, L., J. Francis, R. Islam, et al., A guide to using the Theoretical Domains Framework of behaviour change to investigate implementation problems. Implement Sci, 2017;12(1):77. [↑](#footnote-ref-2)
